# Interpersonal Relatedness and Non-suicidal Self-Injurious Behaviors in Female Adolescents With Borderline Personality Disorder

**DOI:** 10.3389/fpsyt.2021.731629

**Published:** 2021-11-15

**Authors:** Fabian Guénolé, Solène Spiers, Ludovic Gicquel, Veronique Delvenne, Marion Robin, Maurice Corcos, Alexandra Pham-Scottez, Mario Speranza

**Affiliations:** ^1^Centre Hospitalier Universitaire de Caen, Service de Psychiatrie de l'Enfant et de l'Adolescent, Caen, France; ^2^Centre Hospitalier Universitaire de Poitiers, Service de Psychiatrie de l'Enfant et de l'Adolescent, Poitiers, France; ^3^Service de Pédopsychiatrie, Hôpital Universitaire des Enfants Reine Fabiola, Brussels, Belgium; ^4^Institut Mutualiste Montsouris, Service de Psychiatrie de l'Adolescent et de l'Adulte Jeune, Paris, France; ^5^Université Paris-Saclay, UVSQ, INSERM, Centre for Research in Epidemiology and Population Health (CESP), Team “DevPsy,” Villejuif, France; ^6^Hôpital Sainte-Anne, Clinique des Maladies Mentales et de l'Encéphale, Paris, France; ^7^Centre Hospitalier de Versailles, Service Universitaire de Psychiatrie de l'Enfant et de l'Adolescent, Le Chesnay, France

**Keywords:** NSSI deliberate self-harm, adolescents, interpersonal relatedness, borderline personality disorder, depressive experience questionnaire, personality development, self-definition, dependency

## Abstract

**Background:** Psychopathological models of adolescent borderline personality disorder (BPD) suggest that non-suicidal self-injuring (NSSI)—a particularly frequent symptom in girls—may constitute a way of coping with distress resulting from interpersonal concerns they typically experience as a developmental psychopathological feature.

**Objectives:** Our objective was to investigate the relationship in BPD female adolescents between NSSI and the Sidney Blatt two-polarities model of personality development, which focuses on the psychological processes of interpersonal relatedness and self-definition.

**Methods:** The study was conducted within the European Research Network on Borderline Personality Disorder in Adolescence, using the Depressive Experience Questionnaire (DEQ).

**Results:** BPD patients (*n* = 59; mean age = 16.6 ± 1.3) scored significantly higher than healthy controls on the two DEQ sub-factors assessing the more immature forms of Interpersonal Relatedness (*Neediness*) and Self-definition (*Self-criticism*) and significantly lower on the more mature form of Self-definition (*Efficacy*). BPD adolescents with NSSI showed significantly higher scores on both mature and immature forms of Interpersonal Relatedness (*Neediness* and *Connectedness*) compared to BPD adolescents without NSSI. A logistic regression analysis showed that the subfactor *Neediness* of the DEQ was the only significant predictor of the presence of NSSI among BPD adolescents.

**Conclusions:** The preliminary results of this study suggests that NSSI in adolescents with BPD is developmentally linked to high developmental concerns in the domain of interpersonal relatedness, which may be taken into consideration in clinical practice. More studies are necessary to better understand the relationships between NSSI and developmental psychopathology in borderline adolescents.

## Introduction

Nonsuicidal Self-Injury (NSSI), the repeated infliction of injuries to the surface of the body without suicidal intent (1), is part of borderline personality disorder's symptoms (1), mainly in the form of deliberate cutting (with knifes, razors, or broken pieces of glass) (2). NSSI in BPD seems to be associated with severity of symptoms (3), and generally begins before adulthood (4).

BPD is indeed the most frequent mental disorder among inpatient adolescents with NSSI (5, 6), as it is the case in community adolescents with NSSI (7, 8); both types of studies indicating that the phenomenon particularly applies to adolescent girls (8, 9). Some data also suggest that NSSI during adolescence may predict BPD in early adulthood (10–12).

Current research in psychopathology supports the notion that NSSI has two main psychological functions (13, 14): (i) an intrapersonal one used as a mean to regulate emotions; and (ii) an interpersonal one, which serves to communicate with the social environment. The intrapersonal function of NSSI corresponds to the relief of painful emotional tension (13, 14), perhaps by transforming distressing emotions experienced passively into an active and controlled physical pain (15). The interpersonal function is seen as a behavioral way for the individual to communicate distress, verify affection, or encourage caregiving from others (13, 16). This is consistent with the phenomenology of self-injuring in BPD patients, which is typically precipitated and influenced by feelings related to loss, rejection or abandonment (17–20).

One way to address the psychopathological functions of NSSI in patients with borderline personality disorder can be offered by the theoretical perspective proposed by Sidney Blatt of the two central dimensions of personality development: interpersonal relatedness and self-definition (21, 22). This “two-polarities model” (23) is based on the fundamental identification of two types of experiences framed by personality development: the first one focused on concerns associated with disruption in relationships with others (with feelings of loss, abandonment and loneliness) and the second one centered on problems concerning identity (associated with low self-esteem, feelings of failure, culpability, and lack of self-confidence). According to Blatt (23), maladaptive behaviors would emanate directly from an overemphasis and exaggeration of one of the two essential developmental lines of the personality: the Dependent/Anaclitic line, which concerns the establishment of satisfying interpersonal relationships, and the Self-critical/Introjective line, which focuses on the achievement of a positive and cohesive sense of self (23).

Blatt et al. (24) have initially developed the *Depressive Experience Questionnaire* (*DEQ*) to assess these two dimensions which emerge as independent factors in analytic studies. However, subsequent theoretical developments have suggested that different levels could be identified among these dimensions, each following a developmental trajectory from immature to more mature forms of interpersonal relatedness and self-definition. Investigations using the DEQ have thus identified two levels within the Dependency factor (relabelled more appropriately, Interpersonal Concerns): a first sub-factor, labeled Neediness, assesses feelings of loneliness, and insecurity as well as a marked vulnerability to nonspecific experiences of loss, rejection, and abandonment. The second sub-factor, labeled Connectedness, appears to assess a more mature level of interpersonal relatedness, including valuing intimate relationships and being concerned about disruptions of particularly meaningful, specific, and interpersonal relationships (23). In a similar way, the development of a sense of self seems to follow an analogous progression. Research conducted with the DEQ has identified two levels within the development of self-definition: a first level, which corresponds to the Self-critical factor of the DEQ, assesses concerns about self-worth and failure to meet self and externally imposed standards. The second level contains more positive, proactive expressions of competence and confidence in oneself and in the future. Items corresponding to this more mature level of self-definition load mostly on the Efficacy factor already identified within the DEQ. Thus, the DEQ appears to measure maladaptive and more adaptive dimensions of interpersonal relatedness (Neediness and Connectedness), as well as maladaptive and more adaptive dimensions of self-definition (Self-criticism and Efficacy) (23).

The aim of this paper was to preliminary explore the link in adolescent girls with BPD between NSSI and the dimensions of Blatt's model of personality development. We specifically sought to explore if BPD girls with NSSI would present more immature forms of interpersonal relatedness and/or self-definition compared to those without NSSI. Given the emotional and relational features of dependency observed in patients with BPD, its psychopathological association with both intrapersonal and interpersonal functions of NSSI could be expected, both being linked in such patients to feelings of loss, rejection, and abandonment. We thus hypothesized that NSSI would be specifically associated with developmental concerns in the interpersonal relatedness domain.

## Methods

### Participants

The study sample was drawn from a European research study investigating the phenomenology of BPD in adolescence (European Research Network on Borderline Personality Disorder, EURNET BPD) [see Corcos et al. (25) for a full description of the study]. The research network was composed of five university psychiatric centers in France, Belgium, and Switzerland. During the period between January and December 2007, all consecutively admitted adolescents aged 15–19, both in and out-patients, were clinically screened by the consulting psychiatrists to look for a diagnosis of BPD, according to DSM-IV criteria (26). Adolescents meeting a clinical diagnosis of BPD were then referred to the research team for a further assessment and confirmation of the diagnosis of BPD; exclusion criteria were a diagnosis of schizophrenia or any chronic and/or serious medical illness involving vital prognosis.

Adolescents fulfilling the criteria for BPD according to clinicians were further investigated with a diagnostic evaluation of DSM-IV Axis-I and Axis-II disorders and a self-report questionnaire eliciting socio-demographic data and psychopathological features. Diagnosis of BPD was ascertained through the Structured Interview for DSM-IV Personality (SIDP-IV), a semi-structured interview assessing each of the 10 DSM-IV personality disorders, including BPD (27). The reliability and validity of the SIDP-IV have been established in adolescents and young adults and have been validated in French (28). Special attention was paid to the question of the 1-year duration of the symptoms and to the pervasive and persistent nature of the traits.

As the clinical search for the presence of NSSI is part of the SIDP-IV, all patients were systematically questioned on this topic. As indicated in the SIDP-IV, NSSI was defined as a deliberate and severe self-harm behavior such as cutting or burning at the exclusion of superficial behaviors such as scratching, skin picking. Taking inspiration from Zanarini et al. (29), patients were considered with NSSI when they stated having had at least one episode in the past 6 months.

Final diagnoses were established by the best-estimate method on the basis of the interviews and any additional relevant data from the clinical record according to the LEAD standard (30). At the end of the clinical assessment session, an overall level of psychosocial functioning was calculated for each patient according to the Global Assessment of Functioning (GAF) (31).

An advertisement for the study was placed in schools and universities to recruit adolescents for the control group. The procedure for the nonclinical sample was identical to that of the borderline sample. Control participants were screened in order to make sure they did not have a personality disorder according to the SIDP-IV and were excluded if they had a history of or ongoing follow-up with a psychiatrist or a psychologist.

This study was approved by the ethics committee of the Hôtel Dieu Hospital in Paris (authorization n° 0611259). Results were collected in an anonymous database according to the requirements of the French national committee for private freedoms. All participants, adolescents and parents, signed informed consent after receiving a full description of the study, explanation of its purpose, and information about the confidentiality of the data.

### Measures

Blatt's model of personality development (21, 23) was investigated through the *Depressive Experience Questionnaire* (*DEQ*) (24). The *DEQ* is a 66-item self-report scale rated on a 7-point Likert scale ranging from 1 (“strongly disagree”) to 7 (“strongly agree”). The instrument was developed by assembling items describing stable personality characteristics frequently found in patients with depression (23). Factor analyses have yielded three factors matching the constructs of *Interpersonal Relatedness, Self-Criticism*, and *Efficacy* (23). Subsequent factor analyses have identified two sub-factors within the *Interpersonal Relatedness* factor, namely *Neediness* and *Connectedness*, corresponding to different levels of maturation of interpersonal relatedness (23). The *DEQ* has been shown to have high internal consistency, test-retest reliability, convergent and discriminant validity, as well as a high level of construct validity (23). We calculated scores for *Self-Criticism* and *Efficacy* using the factor-weighting procedure provided by Blatt et al. (24) and adapted in French by Atger et al. (32); we also calculated the sub-factor scores for the *Neediness* and *Connectedness* subscales of the *Interpersonal Relatedness* factor using the *ad hoc* scoring program for SPSS (Besser and Babchoock, personal communication).

Symptoms of depression during the last 2 weeks were assessed using the *Adolescent Depression Rating Scale* (*ADRS*), a 10-item self-administered questionnaire with yes/no responses specifically developed to assess depression intensity among adolescents (33).

### Analyses

Statistical comparisons were conducted across BPD and control groups, and across BPD adolescents with and without NSSI, for sociodemographic and clinical characteristics; Student's *t*-tests were used for quantitative variables, and Chi-square tests for qualitative ones. As BPD adolescents with NSSI displayed significantly lower GAF scores (*p* < 0.001) than those without NSSI, we performed an ANCOVA between NSSI groups with GAF as a covariate. Intercorrelations within BPD adolescents for DEQ and GAF scores were calculated using Pearson's coefficient. Finally, to investigate which DEQ sub-factors best predicted the presence of NSSI in BPD adolescents, we performed a logistic regression (NSSI+ vs. NSSI-) analysis with an enter procedure with the four sub-factors of the DEQ and GAF as predictors.

Analyses were performed using SAS software, version 9.4; statistical significance was set at *p* < 0.05.

## Results

The study group comprised 59 BPD adolescent girls (mean age of 16.6 ± 1.3 years) and a control group of 45 healthy adolescent girls (mean age of 16.3 ± 0.9 years). Sociodemographic and clinical characteristics of BPD and control groups are listed in Table 1. Both groups did not significantly differ for sociodemographic characteristics; adolescents with BPD displayed significantly less healthier results for all clinical comparisons, except for the *Connectedness* subscale of the DEQ.

**Table 1:** Sociodemographic and clinical characteristics of BPD and control groups.

**Variables**	**BPD group**	**Control group**	Statistics^*^	
	**(*N* = 59)**	**(*N* = 45)**		
	**M (SD)/*N* (%)**	**M (SD)/*N* (%)**	***t*/*c*^**2**^**	***p***
**Sociodemographic characteristics**				
Age (years)	16.6 (1.3)	16.3 (0.9)	1.74	ns
Educational level			0.41	ns
- High school/college	46 (78%)	39 (87%)		
- Middle school	13 (22%)	6 (13%)		
Parental marital status			1.23	ns
- Married/cohabiting	34 (58%)	33 (74%)		
- Separated	25 (42%)	12 (26%)		
Socio-Professional Status			0.33	ns
- Executives and intellectual professions	36 (61%)	29 (66%)		
- Employee, artisans, and workers	23 (39%)	16 (34%)		
**Clinical characteristics**				
Inpatient care	34 (58%)	–	–	–
Current major depression	20 (34%)	0 (0)	37.5	**0.001**
ADRS	5.8 (2.6)	2.34 (2.8)	6.98	**0.001**
GAF	53.8 (13.3)	94.7 (7.6)	−8.57	**0.001**
NSSI	40 (68%)	0 (0)	61.9	**0.001**
**DEQ interpersonal concern**				
- Neediness	50.0 (9.0)	45.3 (8.5)	4.89	**0.001**
- Connectedness	42.0 (7.6)	40.4 (6.7)	2.78	ns
**DEQ self-definition**				
- Self-criticism	1.09 (0.9)	−0.37 (0.9)	5.58	**0.001**
- Efficacy	−0.39 (0.9)	−0.93 (0.8)	3.21	**0.002**

*ADRS, Adolescent Depression Rating Scale; GAF, General Functioning Assessment; NSSI, Non-Suicidal Self-Injury; DEQ, Depressive Experience Questionnaire; ns, not significant*.

* *Student's t-tests for dimensional variables; Chi-square tests for categorical variables*.

*Bold values are the significant values*.

Among the BPD group, 40 adolescents (67.8%) reported having had at least one episode of NSSI behaviors during the last 6 months. None of the adolescents in the control group reported NSSI behaviors during the last 6 months. Sociodemographic and clinical characteristics of NSSI+ and NSSI– BPD subgroups are listed in Table 2. Both subgroups did not significantly differ for sociodemographic characteristics. BPD adolescents with NSSI had significantly lower GAF scores than BPD adolescents without NSSI (62.3 ± 11.9 vs. 49.5 ± 12.0; *p* < 0.001); proportions of inpatient care, current major depression, and ADRS scores did not significantly differ across subgroups.

**Table 2:** Sociodemographic and clinical characteristics of NSSI+ and NSSI– BPD subgroups.

**Variables**	**NSSI+ BPD subgroup**	**NSSI– BPD subgroup**	Statistics^*^	
	**(*n* = 40)**	**(*n* = 19)**	

	**M (SD)/*****N*** **(%)**	**M (SD)/*****N*** **(%)**	***t*** **/** ***c*** ^**2**^	***P***
**Sociodemographic characteristics**				
Age (years)	16.7 (1.1)	16.5 (1.6)	0.07	ns
Educational level			0.41	ns
- High school/college	31 (77%)	15 (79%)		
- Middle school	9 (22%)	4 (21%)		
Parental marital status			0.76	ns
- Married/cohabiting	25 (62%)	9(47%)		
- Separated	15 (37%)	10 (52%)		
Socio-professional status			0.42	ns
- Executives and intellectual professions	23 (57%)	8 (42%)		
- Employee, artisans, and workers	17 (43%)	11 (58%)		
**Clinical characteristics**				
Inpatient care	25 (62%)	9 (47%)	1.43	ns
Current major depression	16 (40%)	4 (21%)	1.06	ns
ADRS	6.0 (2.6)	5.4 (2.8)	0.29	ns
GAF	49.5 (12.0)	62.3 (11.9)	−8.57	**<0.001**
**DEQ interpersonal concern**				
- Neediness	52.8 (8.2)	45.4 (9.1)	4.63	**<0.05** ^**#**^
- Connectedness	43.8 (7.7)	38.2 (5.7)	9.53	**<0.01** ^**#**^
**DEQ self-definition**				
- Self-criticism	1.17 (1.0)	0.92 (0.9)	0.18	ns^#^
- Efficacy	−0.27 (0.9)	−0.65 (0.9)	1.56	ns^#^

*ADRS, Adolescent Depression Rating Scale; GAF, General Functioning Assessment; NSSI, Non-Suicidal Self-Injury; DEQ, Depressive Experience Questionnaire; ns, not significant*.

* *Student's t-tests for dimensional variables; Chi-square tests for categorical variables*.

# *ANCOVA with GAF as covariable*.

*Bold values are the significant values*.

The ANCOVA with GAF score as a covariate showed that BPD adolescents with NSSI had significantly higher scores on *Neediness* (52.8 ± 8.2 vs. 45.4 ± 9.1; *F* = 4.6; *p* < 0.05) and *Connectedness* (43.8 ± 7.7 vs. 38.2 ± 5.7; *F* = 9.53; *p* < 0.01) compared to BPD adolescents without NSSI; *Self-criticism* and *Efficacy* scores did not significantly differ across subgroups.

Intercorrelations between DEQ and GAF scores within the BPD group and subgroups are listed in Table 3. A significant positive correlation was found in the whole BPD group and in both NSSI+ and NSSI– subgroups between *Neediness* and *Connectedness*. In the whole BPD group a negative correlation was found between *Self-criticism* and GAF scores (*r* = −0.35; *p* < 0.01). In the NSSI+ subgroup a significant positive correlation was found between *Connectedness* and GAF scores (*r* = 0.39; *p* < 0.05) and two significant negative correlations were found, respectively, between *Connectedness* and *Self-criticism* (*r* = −0.35; *p* < 0.05), and between *Self-criticism* and GAF scores (*r* = −0.34; *p* < 0.05). Within the NSSI– BPD subgroup, a negative correlation was found between *Neediness* and GAF scores (*r* = −0.61; *p* < 0.05). Overall, results show that the two DEQ sub-factors assessing the more immature forms of Interpersonal Relatedness (*Neediness*) and Self-definition (*Self-criticism*) were coherently associated with a worst general functioning.

**Table 3:** Pearson's correlation matrices between DEQ and GAF scores within the BPD group, and the NSSI+ and NSSI– BPD subgroups.

	**Neediness**	**Connectedness**	**Self-criticism**	**Efficacy**	**GAF score**
**BPD group (*****n*** **= 59)**					
Neediness	–				
Connectedness	0.72^***^	–			
Self-criticism	−0.16	−0.25	–		
Efficacy	−0.11	−0.03	0.17	–	
GAF score	−0.21	0.03	−0.35^**^	−0.08	–
**NSSI+ BPD subgroup (*****n*** **= 40)**					
Neediness					
Connectedness	0.69^***^	–			
Self-criticism	−0.30	−0.35^*^	–		
Efficacy	−0.27	−0.15	0.23	–	
GAF score	0.23	0.39^*^	−0.34^*^	−0.08	–
**NSSI– BPD subgroup (*****n*** **= 19)**					
Neediness	–				
Connectedness	0.68^**^	–			
Self-criticism	−0.07	−0.23	–		
Efficacy	−0.05	−0.01	−0.03	–	
GAF score	−0.61^**^	−0.28	−0.33	0.16	–

* *p < 0.05*;

** *p < 0.01*;

*** *p < 0.001. BPD, Borderline Personality Disorder; NSSI, Non-Suicidal self-injuring; GAF, General Functioning Assessment; DEQ, Depressive Experience Questionnaire*.

Finally, the logistic regression analysis showed that the subfactor Neediness of the DEQ was the only significant predictor of the presence of NSSI among BPD adolescents [Neediness: Exp(B) = 1.11, *p* = 0.02]. The overall model was significant [−2log L = 53.7, Model F (df = 5) = 12.5, *p* < 002] and explained 37% of the variance (Nagelkerke R^2^ = 0.37; Table 4).

**Table 4:** Prediction of NSSI behaviors by DEQ sub-factors in BPD adolescents.

**Variables**	**B**	**S.E**.	**Wald**	**Sig**.	**Exp(B)**
GAF	−0.21	0.02	0.74	0.39	0.98
Self-criticism	−0.12	0.32	0.14	0.71	0.89
Efficacy	0.70	0.39	3.17	0.75	2.01
Neediness	0.11	0.05	5.17	**0.02**	1.11
Connectedness	−0.72	0.05	1.77	0.18	0.93

*Logistic regression. Method: Enter. Variable to be predicted: BPD with NSSI +. Predictive variables: DEQ sub-factors: Self-criticism, Efficacy, Neediness, Connectedness, GAF. −2logL = 53.7. Modèle F (df = 5) = 14.2, p < 0.014, Nagelkerke R^2^ 0.37. Bold values are the significant values*.

## Discussion

BPD adolescents with NSSI behaviors may represent a specific subgroup of BPD adolescents with a more severe clinical profile and negative outcome. In our sample of BPD adolescents of our study, NSSI behaviors were associated with a poorer global functioning. This is in line with the results of previous works, which have shown that NSSI in BPD adolescents is linked with clinical severity (34, 35). Exploring the psychopathological features associated with NSSI in BPD adolescents is paramount to define specific strategies to take care of this complex population.

In accordance with the main hypothesis of the study, results showed that, among borderline girls, those who expressed NSSI behaviors displayed significantly higher concerns on *Neediness* and *Connectedness* dimensions, but not on *Self-criticism* and *Efficacy* ones, of Blatt's model of personality development (21, 23), independently from general functioning. Moreover, the logistic regression has confirmed that the subfactor Neediness of the DEQ was the only significant predictor of the presence of NSSI among BPD adolescents.

These results suggest that NSSI in adolescents with BPD is associated with concerns within the whole *Interpersonal Relatedness* (IR) polarity of Blatt's two-polarities model of personality development (21, 23). *Neediness* and *Connectedness* levels of IR have in common to reflect a developmental trend for negative emotions arising from relational loss or its apprehensiveness, and to loneliness (23). It could thus be hypothesized that, among borderline adolescents, those with NSSI have the greatest emotional reactivity to relational disruption; this could reflect attachment insecurity, which seems to vary from moderate to severe levels in borderline patients (36).

What distinguishes *Neediness* and *Connectedness* levels within Blatt's developmental model is: (i) the precise nature of emotional concerns around the psychological processing of loss; (ii) the extent of concerned social relationships; and (iii) the repercussions on subjective efficacy. Indeed, while *Neediness* corresponds to intense fear, regarding loss of any relation, with consequent feelings of rejection, helplessness and despair, *Connectedness* involves sadness regarding loss of specific and valued relationships, leading to rather sufferable loneliness. While high *Neediness* is considered a clearly maladaptive pattern involving early developmental issues, high *Connectedness* is considered more developmentally mature, and even somewhat adaptive (23). In this study, BPD adolescents with NSSI showed high scores on *Neediness* and *Connectedness* (77.5 and 72.5% higher than healthy controls on average, respectively) with positive strong correlations between these two sub-factors within BPD group and subgroups, which reflect the commonalties of these dimensions centered on relational issues at different level of maturity. Since *Neediness* is thought to reflect an early maladaptive pattern, we could suggest that it may be the modal variable of the whole IR pattern found in BPD adolescents with NSSI, as suggested by the results of the regression analysis showing that Neediness was the only significant predictor of the presence of NSSI among BPD adolescents. BPD Adolescents with NSSI would thus globally be characterized by higher anaclitic dependency, as it has been suggested in adult patients with BPD (37, 38). This is consistent with the phenomenology of self-injuring in BPD patients, which is typically precipitated and influenced by feelings related to loss, rejection or abandonment (17, 18).

This result also supports both the intrapersonal and interpersonal functions of NSSI behaviors in BPD adolescents (13, 14). According to the theoretical model developed by John Gunderson (39), the interpersonal sensitivity of adolescents with borderline personality disorders may explain a large part of the clinical picture of these patients and helps better understand their clinical heterogeneity. Without dwelling on DSM-5 BPD symptoms that specifically relate to interpersonal difficulties (such as Fear of abandonment and Unstable relationships), most of the other BPD symptoms are directly or indirectly related to the interpersonal hypersensitivity and may be considered as ways to regulate emotions elicited by relationships difficulties or to communicate relational distress to the social environment: criterion 3 describes how identity disturbances are most often manifested in situations where the adolescent experiences a lack of meaningful relationships, support and attention from others; the impulsivity criterion (criterion 4) includes unprotected sex and angry outbursts in the context of relationships; self-harming behaviors (self-harm and suicide attempts) (criterion 5) are often precipitated by threats of separation or rejection; mood reactivity or emotional instability (criterion 6) often reflects the adolescent's extreme reactivity to interpersonal tensions; intense and inappropriate anger (criterion 8) is often triggered by the adolescent feeling neglected or abandoned by others. Finally, the dissociative symptoms of criterion 9, apparently the furthest from the interpersonal dimension, are currently at the heart of major developments in the etiopathogenic theories of BPD, which consider these symptoms as adaptive phenomena in the face of relational stressors in contexts of attachment desorganisation (40, 41). The oscillations in the symptomatic expression that we observe in BPD adolescents correspond to their different modalities of response to the perceived availability of significant others. When an adolescent with BPD feels connected to someone, he or she may idealize the other person. Although anxious, he/she is receptive to the help that is brought to him/her. His/her situation remains fragile, however, and characterized by a hyper-vigilance to any sign of possible abandonment or rejection. When, inevitably, the adolescent perceives such signs of rejection, this state of connection turns into a so-called threatened state, which leads the adolescent with BPD to express intense rage and eventually to engage in self-damaging acts. The use of self-injurious behaviors could then be considered as an extreme solution adopted by these adolescents to regulate their negative emotions, especially as they present difficulties in identifying and expressing emotions (alexithymia), as it has been described in the literature (42–45). However, NSSI behaviors lead the relationship partners to withdraw from the relation, leaving the adolescent with BPD truly alone (isolated state). Since the adolescent with BPD cannot tolerate solitude, he or she may exhibit dissociative symptoms (39) or increase NSSI behaviors in a downward spiral.

Finally, we confirmed that, as a group, borderline adolescent girls score high on both psychopathological axes of Blatt's model of personality development (46, 47), thus placing adolescent BPD in the anaclitic and introjective “mixed group” of Blatt's two-polarities model (48, 49).

This study entails several limits, which must be stressed in order to well-appreciate the significance and applicability of results. First, the small sample sizes, particularly regarding patients without NSSI, may have limited the validity of the results, which should thus be considered as preliminary and in need to be replicated in larger clinical samples. Second, the significant correlation observed between Neediness and Connectedness scores in patients may have reduced the power of regression analyses; this in addition to the limited statistical power of the analysis, it is possible that the significance of Connectedness may have been underestimated in the model. We also wish to emphasize that the study does not make it possible to judge whether the results observed are specific to the population studied, or could be found for other mental disorders. Finally, it can be stressed that the results presented here are secondary analyses from the EURNET BPD, acquired several years ago. Although no study suggests that BPD phenomenology in adolescents has significantly evolved in recent years, the results may not fully correspond to current patients.

To conclude, the preliminary results of this study suggests that NSSI in adolescents with BPD is linked to high interpersonal relatedness developmental concerns, which may be taken into consideration in clinical practice. More studies are necessary to better understand the relationships between NSSI and developmental psychopathology in borderline adolescents.

## Data Availability Statement

The raw data supporting the conclusions of this article will be made available by the authors, without undue reservation.

## Ethics Statement

The study was approved by the Ethics Committee of the Hôtel Dieu Hospital in Paris (authorization n° 0611259). Written informed consent to participate in this study was provided by the participants' legal guardian/next of kin.

## Author Contributions

FG participated in data analysis and interpretation and in writing the manuscript. MS, MC, and AP-S initiated and designed the protocol, collected data, participated in data analysis, and interpretation and in revising the manuscript. SS, LG, MR, and VD participated in data analysis and interpretation and in revising the manuscript. All authors read and approved the final manuscript and contributed sufficiently to the project to be included as authors.

## Funding

This research was supported by a grant from the Wyeth Foundation for Child and Adolescent Health and by a grant from the Lilly Foundation. Both had no role in conception and design of the article, in the writing of the manuscript, and in the decision to submit the paper for publication.

## Conflict of Interest

The authors declare that the research was conducted in the absence of any commercial or financial relationships that could be construed as a potential conflict of interest.

## Publisher's Note

All claims expressed in this article are solely those of the authors and do not necessarily represent those of their affiliated organizations, or those of the publisher, the editors and the reviewers. Any product that may be evaluated in this article, or claim that may be made by its manufacturer, is not guaranteed or endorsed by the publisher.
